# Chloroform

**Published:** 1865-08

**Authors:** 


### CHLOROFORM.

Chloroform, when administered by inhalation during the period of Menstruation, Dr. Kidd affirms, may have the effect ot inducing the belief that an assault has been attempted in a criminal way, whilst under its influence. Now, although we can not, from our own experience, connect with certainty the fact of Menstruation with this effect in more than a single instance, we are cognizant of three well-marked cases of the kind occurring in this city, and rumor speaks of several others. We were well acquainted with an elderly gentleman whose wife was so firmly convinced that a dentist had endeavored to take improper liberties with her whilst under the influence of chloroform, that he had much difficulty in convincing her that her husband had not left her side during the whole time. We also knew of a young girl who, after an important operation, during which this anæsthetic was administered, positively affirmed that an attempt had been made upon her chastity by the chief surgeon; and from which trouble might have arisen had not other surgeons been present, and her friends been in the adjoining room during its performance.

The third, a case well known to the profession, in which a respectable woman, whilst menstruating, was put under the influence of chloroform for the abstraction of a tooth, when she afterwards suffered so strongly from a similar illusion that the husband being fully persuaded of its truthfulness, caused the prosecution and imprisonment of the dentist for assault. He was acquitted of the crime, but received a reprimand from the judge for having administered an anæsthetic without the presence of witnesses.

This case elicited much comment at the time, and has had the effect ever since of rendering our physicians more than ordinarily cautious in the employment of chloroform in the absence of the patient's own friends.—*Canada Lancet.*

